# RAFA: RNA all-atom structure reconstruction from sparse anchor coordinates

**DOI:** 10.1093/bioinformatics/btag746

**Published:** 2026-10-06

**Authors:** Lei Wang, Zilu Zeng

**Affiliations:** State Key Laboratory of Green Pesticide, International Joint Research Center for Intelligent Biosensor Technology and Health, Central China Normal University, Wuhan 430079, China; School of Software Engineering, Huazhong University of Science and Technology, Wuhan 430074, China; Wuhan Children’s Hospital, Tongji Medical College, Huazhong University of Science and Technology, Wuhan 430070, China

## Abstract

**Summary:**

We present RAFA, a C++ command-line tool for RNA all-atom structure reconstruction from sparse or partial coordinates. RAFA targets settings in which coarse-grained modelling, low-resolution fitting, or partial experimental interpretation provides RNA anchor atoms but not a complete atomic model. The method retrieves experimentally observed 3–5 nt all-atom RNA fragments, fits them to local anchors, combines overlapping fragment-derived coordinate estimates by weighted consensus, and routes sparse and rich inputs through distinct reconstruction paths. In an independent RNA3DB train/test evaluation, no library–test pair exceeded 80% global sequence identity. RAFA achieved lower all-atom root-mean-square deviation (RMSD) values than Arena in 73.3% of target–mode comparisons, with the largest gains in input modes with the fewest structural constraints.

**Availability and implementation:**

RAFA is available at https://github.com/wangleiofficial/RAFA and archived at https://doi.org/10.5281/zenodo.20951205.

## 1 Introduction

RNA function depends strongly on three-dimensional structure, and atomically detailed models remain the most informative representation for analysing folding, catalysis, ligand recognition, and steric interactions (Miao and Westhof 2017, Zhang *et al*. 2022). In practice, however, many experimental and computational pipelines produce incomplete all-atom RNA models. Cryo-EM interpretation, low-resolution fitting, coarse-grained simulation, and intermediate outputs from structure-generation workflows may provide only phosphate, ribose, backbone, or base anchors. Converting such partial coordinate models into full all-atom structures is therefore a practical reconstruction problem.

RAFA addresses a different task from de novo RNA structure prediction. Established sequence- and restraint-driven approaches include FARNA/FARFAR, MC-Fold/MC-Sym, iFoldRNA, ModeRNA, RNAComposer, 3dRNA, and FARFAR2 (Das and Baker 2007, Parisien and Major 2008, Sharma *et al*. 2008, Rother *et al*. 2011, Popenda *et al*. 2012, Zhao *et al*. 2012, Watkins *et al*. 2020). Coarse-grained and learning-based approaches include NAST-like modelling, SimRNA, trRosettaRNA, and deep geometrical-potential models (Jonikas *et al*. 2009b, Boniecki *et al*. 2016, Li *et al*. 2023, Wang *et al*. 2023). RAFA instead starts from an existing partial coordinate model and restores missing atomic detail while preserving the observed anchors.

RNA backmapping, completion, and refinement tools also differ in input requirements. C2A instantiates atoms from reference fragments after secondary-structure decomposition, ABC2A focuses on predefined coarse-grained representations, and QRNAS refines nucleic-acid geometry after model generation (Jonikas *et al*. 2009a, Stasiewicz *et al*. 2019, Shi *et al*. 2024). PDBFixer from the OpenMM ecosystem is mainly suited to nearly complete structures (Eastman *et al*. 2017). These assumptions motivate an automated reconstruction method for heterogeneous sparse RNA anchors.

The primary task-matched baseline is Arena, which reconstructs all-atom RNA from sparse coordinates without user-supplied templates or segment annotations (Perry *et al*. 2023). RAFA differs from Arena in how local structural information is obtained and aggregated. Arena initializes local nucleotide geometry from idealized templates and applies iterative geometric correction. RAFA (*R*NA *A*ll-atom *F*ragment *A*ssembly) retrieves experimentally observed all-atom RNA fragments, fits multiple local candidates to the observed anchors, and combines overlapping fragment-derived atomic-coordinate estimates by weighted consensus. This design exploits short-range backbone–base correlations from known RNA structures when the input is too sparse for direct atom completion.

## 2 Methods

### 2.1 Reconstruction workflow

RAFA is implemented in C++ and provides four command-line entry points: fit-library, dedup-library, rebuild, and rebuild-batch. During reconstruction, RAFA first normalizes the input PDB into ordered canonical RNA residues, canonicalizes standard atom names, and records the observed anchor atoms in each residue. The default auto mode then routes each chain to one of two reconstruction paths. Sparse inputs are handled by fragment reconstruction. Dense ribose or backbone-base inputs are routed to local atom completion because these anchors already constrain much of the local nucleotide geometry.

The fragment-reconstruction path is RAFA’s central algorithmic component. It enumerates all overlapping windows of length 5, 4, and 3 nt. Each window defines a query consisting of its sequence, all observed standard atoms, and any complete P, C4${\;}^{\prime }$, C3${\;}^{\prime }$, or glycosidic-N tracks (N9 for purines and N1 for pyrimidines). Candidate all-atom fragments are retrieved from prebuilt libraries by a hierarchy of exact sequence, central nucleotide, fragment length, coarse-geometry signatures, and rough geometric keys. Each candidate is least-squares fitted to the observed anchors, or to the best available coarse mask when too few exact shared atoms are available. Candidates are scored by fitted root-mean-square deviation (RMSD), local sequence compatibility, missing-anchor penalties, and library bias. RAFA therefore does not use whole-chain sequence identity during inference, but it is not sequence-independent because local sequence contributes to fragment retrieval and scoring.

RAFA then assembles the final model by consensus rather than by selecting one fragment per window. The top candidates from every overlapping window contribute transformed atom coordinates to a residue- and atom-specific accumulator. Each contribution is weighted by exp(−2×score) and by a window-position weight that favours central residues. Existing input atoms are restored after reconstruction, missing atoms are filled from the weighted consensus, and shifted ideal-residue templates are used only when no fragment-derived estimate is available. Post-assembly refinement repairs local bond geometry, base conformation, and close contacts; optional CSSR-derived base-pair information can be used during completion and refinement (Zhang and Pyle 2022). Additional algorithmic details and a stepwise algorithm summary are provided in Supplementary Methods and Table S1, available as supplementary data at *Bioinformatics* online.

RAFA can load the generic core library alone or combine it with one user-selected expert library. In the category-informed benchmark, the externally supplied target label selected the matching rRNA, riboswitch, or aptamer library; core targets used the core library alone. RAFA never searched all three expert libraries simultaneously for a target. Candidates from the core and expert libraries were ranked using the same scoring function, without an additional score bonus or penalty for expert-library candidates. RAFA does not currently infer target categories automatically, so core-only retrieval is the default category-independent setting.

The present implementation and benchmarks are limited to RNA-only inputs. General HETATM context, including ligands and ions, is not used during reconstruction and is not guaranteed to be retained in the output. Fragment retrieval and assembly are performed separately for each RNA chain. Direct RNA–RNA interchain contacts are not favourable retrieval or assembly terms, although final clash cleanup examines the complete multichain model.

### 2.2 RNA 3D Hub-derived libraries

The released libraries were generated from the RNA 3D Hub RNA nonredundant representative set, release 4.29 at the 4.0 Å resolution cutoff (Leontis and Zirbel 2012). The source list contained 3708 nonredundant equivalence classes. Multiple representative classes can originate from the same deposited structure; these classes corresponded to 3072 distinct PDB IDs. Representative RNA chains could be parsed for 3653 classes and were used for fragment-library construction. Representative chains were grouped by category (core, rrna, riboswitch, and aptamer), converted into contiguous canonical RNA fragments of length 3–5 nt, and serialized as binary libraries. Deduplication uses quantized coarse-geometry signatures with default settings—dedup-quant 0.25 and—max-per-signature 1.

For the equivalence-class-excluded release used in the original benchmark, the 72 selected RNA 3D Hub equivalence-class representatives were omitted before library construction. This procedure generated four deduplicated binary libraries: a core library with 4 56 481 fragments, an rRNA library with 9 54 081 fragments, a riboswitch library with 24 356 fragments, and an aptamer library with 1993 fragments. The core library is used for the generic RAFA baseline, whereas one matching category-specific library is enabled in the category-informed core + expert configuration. Because RNA 3D Hub equivalence classes, rather than complete PDB entries, were the exclusion unit, this original split should not be interpreted as PDB-level independent; the overlap analysis is reported in Supplementary Methods and Table S2, available as supplementary data at *Bioinformatics* online.

### 2.3 Benchmark data and evaluation

The original RNA 3D Hub benchmark was generated from the curated representative-chain pool described above (Leontis and Zirbel 2012). The test set contains 72 targets from 67 distinct PDB entries: 44 core RNAs, 11 rRNAs, 9 riboswitches, and 8 aptamers, with 57 short, 7 medium, and 8 long targets. Fifty-four targets are single-chain structures, 17 contain two RNA chains, and one contains three RNA chains. The selected equivalence-class representative was removed before construction of its benchmark library. A retrospective PDB-level analysis nevertheless identified ten additional equivalence-class representatives from five test PDB entries in the source pool (Table S2, available as supplementary data at *Bioinformatics* online). We therefore refer to this split as *equivalence-class-excluded*, not fully structure-independent. For each target, an all-atom reference structure and 11 partial-input variants were generated: P_only, phosphate_only, ribose_only, backbone_noP, backbone_only, base_only, P_C4, P_C4_N, glycoN_only, P_ribose, and P_C1_base.

To test generalization under a stricter split, we independently rebuilt the RAFA core library from the official RNA3DB training split and evaluated a benchmark panel derived from the official test split (Szikszai *et al*. 2024). After retaining one representative per 99%-identity cluster, the official test split contained 660 representatives. Before evaluating either method, we retained 275 single-chain representatives with complete residue-level coordinate coverage and at least 90% canonical heavy-atom coverage. We also excluded 30 training representatives with greater than 80% global sequence identity to any member of this panel. The resulting library contained 23 48 766 deduplicated 3–5-nt fragments from 1509 training representatives (3871 contiguous coordinate segments; 8 46 844 observed nucleotides). An exhaustive global alignment analysis of all 4 14 975 final train–test pairs found no identity above 80%; the maximum was exactly 80.0%. Global identity was computed by Needleman–Wunsch alignment as matches divided by full alignment length, including mismatches and gaps. The train–test separation therefore applies specifically to RAFA’s fragment-library source; Arena was evaluated on the same test panel without modification.

Comparisons against Arena used identical partial inputs and reference structures. On the original benchmark, RAFA was evaluated in core-only and category-informed core + expert modes; the independent RNA3DB experiment used its newly built core library. The default parameters top-k 3, post-iters 8, anchor-mode auto, and use-cssr 1 were fixed before analysis and were not tuned per target or input mode. One Kabsch transformation was fitted per target across all shared P, C4${\;}^{\prime }$, and glycosidic-N anchors and then applied to every predicted atom. All-atom RMSD and mean absolute error (MAE) were computed over standard canonical RNA heavy atoms with matching normalized names in corresponding residues; missing atoms were excluded. Residue coverage is the number of reference residues represented in the scored model divided by the number in the reference. Multichain targets were scored as one structure under one transform, rather than averaged chain by chain. A clash was counted when the interatomic distance was below the sum of van der Waals radii minus 0.401 Å, excluding atoms in the same residue and specified adjacent-residue covalent neighborhoods; interchain pairs were included. Full atom selections and exclusions are provided in Supplementary Methods, available as supplementary data at *Bioinformatics* online.

Inferential comparisons were performed within each input mode using two-sided paired Wilcoxon signed-rank tests, with Holm correction across the 11 modes. Differences were defined as RAFA minus Arena, so negative values favour RAFA. Pooled counts and means across input modes are reported only as descriptive summaries because the same targets recur in every mode. PDBFixer (Eastman *et al*. 2017) was evaluated as a generic completion baseline; sparse inputs frequently did not yield comparable scored outputs, so these results are reported separately in Table S5, available as supplementary data at *Bioinformatics* online.

## 3 Benchmark results

RAFA was evaluated on the original equivalence-class-excluded benchmark and the independent RNA3DB split (Supplementary Tables S3 and S4, available as supplementary data at *Bioinformatics* online). The original benchmark contains 72 targets and 11 partial-input variants per target, for 792 reconstructions per method. Mean residue coverage was effectively identical across Arena and RAFA (overall 0.989), so differences mainly reflect reconstruction accuracy and steric quality rather than reconstruction failure.

Across all 11 original input modes, category-informed RAFA had a lower mean all-atom RMSD than Arena in seven modes and a lower target-level RMSD in 564 of 792 descriptive pairs. The strongest gains occurred for P-only, phosphate-only, P + C4${\;}^{\prime }$, and glycosidic-N-only inputs: RAFA was lower in 245 of 288 pairs and reduced the descriptive mean RMSD from 2.221 to 1.397 Å (Fig. 1). Per-mode paired analyses confirmed lower RMSD for RAFA after Holm correction for these four modes (adjusted *P* from $9.38\times 10^{-11}$ to $1.01\times 10^{-6}$; Table S3, available as supplementary data at *Bioinformatics* online). In contrast, the P + C4${\;}^{\prime }$+N mode illustrates why paired distributions, not pooled means alone, are required: RAFA had a lower median difference (−0.048 Å) and was lower for 50 of 72 targets, but two large positive differences reversed the mean (+0.146 Å).

**Figure 1:**
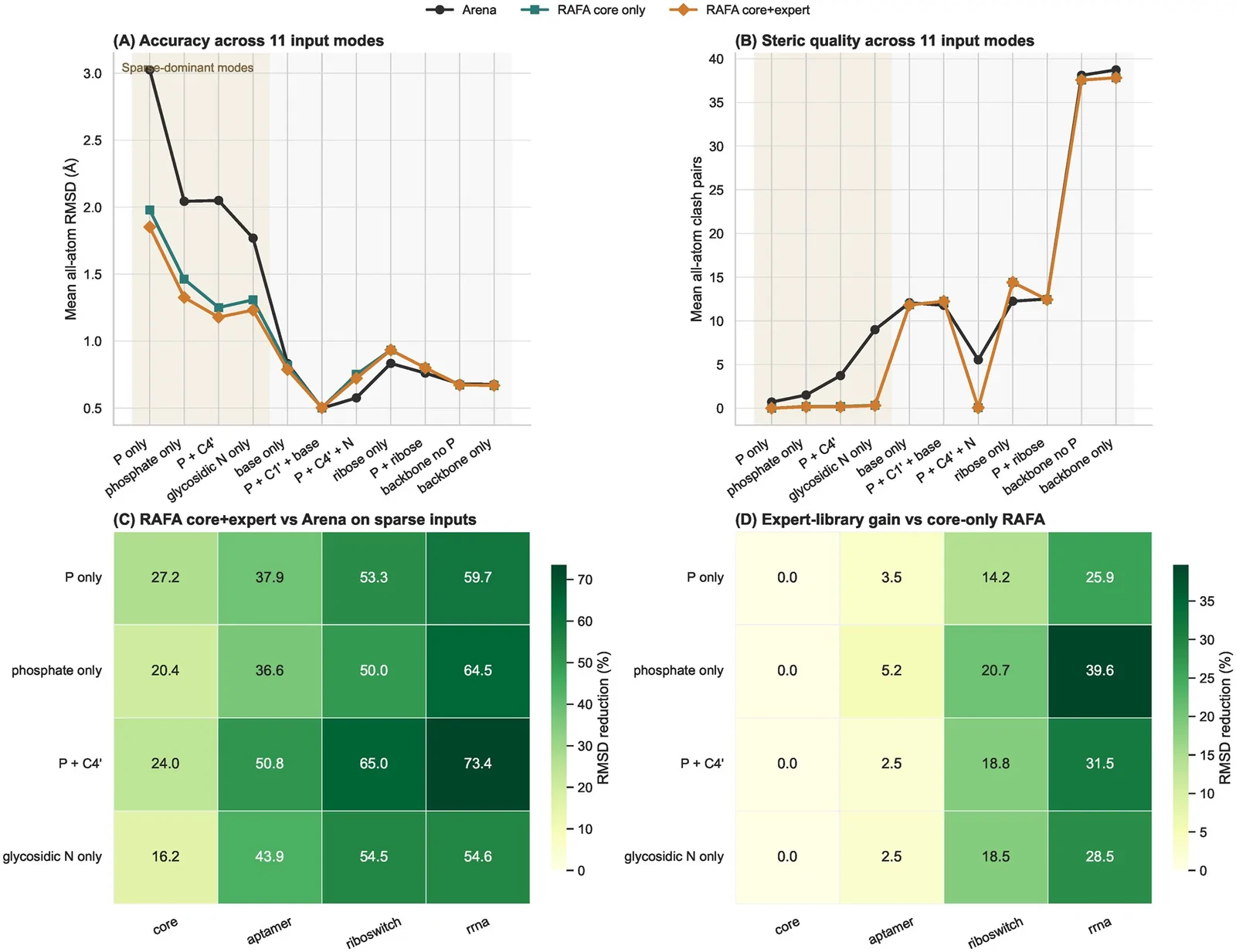
Original RNA 3D Hub equivalence-class-excluded benchmark comprising 72 targets and 11 input modes. (A) Mean all-atom RMSD. (B) Mean all-atom clash-pair count. Shading in A and B denotes the four sparse-dominant modes. (C) Percentage reduction in mean all-atom RMSD for category-informed RAFA relative to Arena across sparse input modes, stratified by target category (core, n=44; aptamer, n=8; riboswitch, n=9; rRNA, n=11). (D) Additional percentage reduction obtained by adding one category-matched expert library to the core-only RAFA configuration. Category-stratified values are descriptive.

The independent RNA3DB experiment reproduced the input-regime dependence under a train/test split with no global sequence identity above 80%. Across all 11 modes, RAFA had a lower descriptive mean RMSD in seven modes and was lower in 2217 of 3025 target-mode pairs. In the five weakly constrained modes (P-only, phosphate-only, P + C4${\;}^{\prime }$, glycosidic-N-only, and base-only), it was lower in 1338 of 1375 pairs and reduced mean RMSD from 2.522 to 1.656 Å. Arena retained lower means for ribose-only, P + C4${\;}^{\prime }$+N, and P + C1${\;}^{\prime }$+base inputs, and was slightly lower on average for P + ribose inputs. Complete paired statistics are provided in Table S4, available as supplementary data at *Bioinformatics* online.

On the original benchmark, expert libraries provided the largest apparent benefit for non-core sparse reconstructions. Relative to the core library alone, category-informed core + expert reduced sparse-mode RMSD by 6.4%–9.3% across the four sparse modes and reached a 39.6% reduction for the 11 rRNA phosphate-only targets (Fig. 1D). These category-stratified estimates are descriptive because the rRNA, riboswitch, and aptamer groups contain only 11, 9, and 8 targets, respectively. Three P-only structural superpositions illustrate local reconstruction differences for targets from the core, riboswitch, and aptamer categories (Fig. S2, available as supplementary data at *Bioinformatics* online).

Median per-target runtimes across input modes were 0.61 s for Arena and 5.06 s for category-informed RAFA. Figure S3, available as supplementary data at *Bioinformatics* online reports both mean and median runtimes. Arena was fast for most targets but required substantially longer runtimes for several large RNA targets, which increased its mean runtime. RAFA had a higher typical recorded cost, and its sparse-input accuracy gains therefore involve a computational trade-off. Runtime measurements are described in Supplementary Methods, available as supplementary data at *Bioinformatics* online.

## 4 Discussion

RAFA was most effective for sparse or heterogeneous partial-coordinate inputs, particularly P-only, phosphate-only, P + C4${\;}^{\prime }$, and glycosidic-N-only modes. Its fragment-library search, fitting of multiple candidates, coordinate consensus, and geometric refinement require more computation than Arena. These additional steps are most useful when few structural anchors are available. RAFA is therefore most suitable for sparse-anchor reconstruction, whereas Arena or RAFA’s local-completion path may be more appropriate when dense ribose and base information is already available or when runtime is a priority.

The RNA3DB evaluation showed that these sparse-input improvements persisted when no library–test pair exceeded 80% global sequence identity. Because local sequence still contributes to fragment retrieval and scoring, RAFA should not be described as sequence-independent. The category-informed results suggest that a matching expert library can improve reconstruction for some RNA classes, although the category subsets were small. Selecting an expert library requires prior knowledge of the target class, and core-only retrieval remains the default when this information is unavailable. The independent RNA3DB panel contained only single-chain canonical RNAs. Multimeric interfaces, modified nucleotides, and structures containing ligands or ions remain to be evaluated.

## Supplementary Material

btag746_Supplementary_Data

## Data Availability

RAFA is available as open-source software at https://github.com/wangleiofficial/RAFA. The archived software snapshot used for this manuscript is available at https://doi.org/10.5281/zenodo.20951205. The repository contains the C++ source code, benchmark drivers, evaluation scripts, example inputs, and workflows for rebuilding fragment libraries from RNA 3D Hub and RNA3DB representatives. Prebuilt equivalence-class-excluded fragment libraries are hosted at https://huggingface.co/datasets/wangleiofficial/RAFA-rna3dhub-libraries. The standard Conda installation command is conda install -c conda-forge -c isyslab-hust rafa.
