# Supplementary material for: RAFA: RNA all-atom structure reconstruction from sparse anchor coordinates
Source: Bioinformatics. 2026 Oct 6;42(10):btag746. doi: 10.1093/bioinformatics/btag746 (PMC13677064; doi:10.1093/bioinformatics/btag746)
Supplement: Supplementary file 1 [file btag746_supplementary_data.pdf]

Supplementary Methods

RNA 3D Hub source set and original benchmark split

The fragment libraries and original benchmark were derived from the RNA 3D Hub RNA nonredundant representative set, release 4.29 at the 4.0 Å resolution cutoff. The source list contained 3708 equivalence classes corresponding to 3072 distinct PDB IDs. Representative RNA chains could be parsed for 3653 classes, which defined the source pool: 3228 core RNA, 292 rRNA, 107 riboswitch, and 26 aptamer entries.

The benchmark selected 72 equivalence-class representatives from 67 distinct PDB entries. It contained 44 core RNAs, 11 rRNAs, 9 riboswitches, and 8 aptamers, and covered 57 short targets (≤120 nt), 7 medium targets (121–400 nt), and 8 long targets (>400 nt). Fifty-four targets contained one RNA chain, 17 contained two chains, and one contained three chains. The selected equivalence-class representative was removed before rebuilding its benchmark library, leaving 3184 core RNA, 281 rRNA, 98 riboswitch, and 18 aptamer representatives.

This exclusion unit requires qualification. RNA 3D Hub is nonredundant at the equivalence-class level, but multiple equivalence classes can originate from one deposited structure. Cross-referencing the 67 test PDB IDs against the staged source catalog identified ten additional equivalence-class representatives from five test PDB entries (Table S2). Thus, the original split is accurately described as *equivalence-class-excluded*; it is not PDB-level independent. We retain the original benchmark because it contains multichain targets and permits comparison with the original analysis. The RNA3DB experiment below provides an independent evaluation in which no library–test pair exceeds 80% global sequence identity.

RNA3DB train/test reconstruction experiment

We downloaded the official RNA3DB 2026-01-05 full release and retained one representative per 99%-sequence-identity cluster in the training and test splits. This procedure yielded 660 representatives in the official test split. Before evaluating either method, we selected a 275-chain benchmark panel by requiring complete residue-level coordinate coverage and at least 90% canonical heavy-atom coverage. The selection criteria were based only on reference-structure completeness and were applied without inspecting RAFA or Arena performance. All 660 test representatives and the final 275-chain panel are recorded in the released manifests.

Training representatives were screened against the 275-chain benchmark panel by global Needleman–Wunsch alignment implemented with Edlib. Identity was defined as exact matches divided by the complete alignment length, including mismatches and gaps. The exclusion operator was strictly greater than 80%; consequently, pairs at exactly 80.0% were retained. Thirty initially selected training representatives exceeded the threshold and were removed.

The final training set comprised 1509 representatives. Coordinate-bearing canonical RNA was split at chain discontinuities into 3871 contiguous segments of at least 3 nt, representing 846 844 observed nucleotides. The same 3–5-nt extraction and deduplication procedure used for RAFA generated 2 348 766 fragments. The final 275-chain test panel spanned 32–730 nt (median, 56 nt; 20 691 nt in total) and 28 RNA3DB structural components. Exhaustive comparison of all 414 975 final train–test pairs found no identity above 80%. The maximum was exactly 80.0%: 32 matches over an alignment length of 40 for test chain 7yed_T and training chain 8fjl_a6.

For each RNA3DB target, the same 11 partial-coordinate modes were generated and supplied to RAFA and Arena. RAFA used only the newly built RNA3DB training library. The train–test identity control therefore applies specifically to RAFA's fragment-library source, while Arena was run unchanged as the external baseline. RNA3DB test entries are single-chain, so the experiment does not test multimeric interchain reconstruction.

Fragment retrieval and coordinate consensus

RAFA represents each input chain as an ordered list of canonical RNA residues and records the observed standard atoms in each residue. The sparse route scans overlapping 5-, 4-, and 3-nt windows. Each query contains its local sequence, all observed standard atoms, and any complete P, C4′, C3′, or glycosidic-N tracks.

Candidate all-atom fragments are retrieved by a hierarchy of exact local-sequence, central-nucleotide, length, coarse-geometry-signature, and rough-geometric-key indexes. A candidate is least-squares fitted to shared anchors, or to the best available coarse mask if too few exact shared atoms are present. The score combines fitted root-mean-square deviation (RMSD), local sequence compatibility, missing-anchor penalties, and library bias. In particular, the implementation uses exact local-sequence buckets where available and includes a local Hamming penalty in the score. RAFA therefore does not use whole-chain global identity during inference, but it is not sequence independent.

RAFA does not select one local fragment as the final answer. The top candidates from every overlapping window contribute transformed atomic coordinates to residue- and atom-specific accumulators. Contributions are weighted by $\exp(-2s)$, where s is the candidate score, and by a window-position factor favouring central residues. Observed input atoms are restored, missing atoms are filled by weighted coordinate consensus, and an aligned ideal-residue template is used only when no fragment-derived estimate is available. The complete sequence of operations is given in Table S1 and illustrated in Fig. S1.

Routing, expert libraries, and defaults

The `auto` route sends sparse inputs to fragment reconstruction and dense ribose/backbone–base inputs to local completion. The generic configuration loads the core library only. In the category-informed original benchmark, the category was read from the externally supplied target manifest: core targets used the core library alone, whereas rRNA, riboswitch, and aptamer targets used the core library plus exactly one matching expert library. RAFA never searched all three expert libraries simultaneously for a target. Candidates from the core and expert libraries were ranked using the same scoring function, without an additional score bonus or penalty for expert-library candidates. RAFA does not automatically classify target category; core-only is the category-independent default.

The benchmark parameters were fixed before analysis: `top-k 3`, `post-iters 8`, `anchor-mode auto`, and `use-cssr 1`. They were not tuned per target or input mode.

Current molecular scope

The parser used in these experiments reads canonical RNA from `ATOM` records. General `HETATM` context, including ligands and ions, is neither used in retrieval nor guaranteed to be retained. Fragment retrieval and coordinate consensus are performed independently for each RNA chain. No favourable interchain-contact term is used during retrieval or assembly, although final clash cleanup evaluates the complete multichain model and includes interchain atom pairs. These constraints limit claims about modified nucleotides, ligand-bound states, and multimer interfaces.

Evaluation definitions

Residues were matched by normalized chain/residue identifiers when available and otherwise by order under the benchmark's deterministic matching procedure. For each target, one Kabsch transform was fitted over all matched P, C4′, and glycosidic-N anchor coordinates and applied to the entire predicted structure. Multichain targets were therefore evaluated under one target-level transform, not by averaging separately aligned chains.

All-atom RMSD was computed over the intersection of normalized atom names for standard canonical RNA heavy atoms in matched residues:

$$\mathrm{RMSD} = \sqrt{\frac{1}{N}\sum_{i=1}^{N}\|\mathbf{x}_i - \mathbf{y}_i\|_2^2}.$$

All-atom mean absolute error (MAE) is the mean absolute Cartesian coordinate difference over the same pairs. Missing atoms are excluded rather than imputed for scoring. The previously named atom-coverage field is residue coverage: matched reference residues divided by all reference residues.

A clash was counted when two scored atoms were closer than the sum of their van der Waals radii minus 0.401 Å. Same-residue pairs were excluded. For adjacent residues, the expected covalent/near-covalent pairs O3′–P, O3′–OP1, O3′–OP2, O3′–O5′, C3′–P, and C4′–P were excluded. Interchain atom pairs were retained in clash evaluation.

Statistical analysis

For each input mode, the primary unit was the target. We defined $\Delta = \mathrm{RMSD}_{\mathrm{RAFA}} - \mathrm{RMSD}_{\mathrm{Arena}}$, so negative values favour RAFA. Two-sided paired Wilcoxon signed-rank tests were used because a directional alternative had not been preregistered. P values were adjusted by the Holm procedure across the 11 modes within each benchmark. Tables report the paired mean and median Δ, interquartile range (IQR), RAFA-lower/tie/Arena-lower counts, and adjusted P value. Aggregates over 792 or 3025 target–mode pairs are descriptive only because each target occurs in 11 modes.

Runtime measurement and interpretation

The compute environment and commands are documented in the GitHub README. Runs used Ubuntu 22.04.5 LTS with 128 logical processor cores, 503 GiB of memory, GCC/G++ 11.4.0, and four benchmark workers.

The recorded runtimes included library loading, preprocessing, reconstruction, refinement, and output writing. Median per-target runtimes across input modes were 0.61 s for Arena and 5.06 s for category-informed RAFA. Figure S3 reports both per-mode means and medians. Arena was fast for most targets but required substantially longer runtimes for several large RNA targets, which increased its mean runtime. All 72 targets were retained.

Supplementary Figures

Supplementary Table S1: Pseudocode for RAFA all-atom structure reconstruction in default `auto` mode.

Algorithm 1 RAFA all-atom structure reconstruction

```
def Reconstruct($X, \mathcal{L}, K = 3, n_{\text{refine}} = 8, \text{use\_cssr} = 1$):
  $X \leftarrow$ NormalizeCanonicalRNA($X$)
  if HasDenseLocalConstraints($X$) then
      $Y \leftarrow$ LocalCompletion($X$)
  else
      $S_{r,a}, W_{r,a} \leftarrow 0, 0$ for every residue $r$ and standard atom $a$    # coordinate and weight accumulators
      for all window lengths $\ell \in \{5, 4, 3\}$ do
          for all contiguous windows $q = X[i : i + \ell - 1]$ do
              $F \leftarrow$ Retrieve($\mathcal{L}$, sequence($q$), anchors($q$), coarse_tracks($q$))
              $C \leftarrow \emptyset$
              for all fragments $f \in F$ do
                  $T_f \leftarrow$ RigidFit($f$, anchors($q$))    # shared anchors or best coarse mask
                  $s_f \leftarrow$ Score($T_f(f), q$)    # fit, local sequence, missing anchors, library bias
                  $C \leftarrow C \cup \{(f, T_f, s_f)\}$
              end for
              for all $(f, T_f, s_f) \in$ LowestScore$_K(C)$ do
                  for all fragment positions $j$ and atoms $a$ in $f$ do
                      $w \leftarrow \exp(-2 s_f)\, p_j$    # $p_j$ favours central window positions
                      $S_{i+j,a} \leftarrow S_{i+j,a} + w\, T_f(\mathbf{x}_{f,j,a})$
                      $W_{i+j,a} \leftarrow W_{i+j,a} + w$
                  end for
              end for
          end for
      end for
      $Y_{r,a} \leftarrow S_{r,a} / W_{r,a}$ wherever $W_{r,a} > 0$    # weighted coordinate consensus
      $Y \leftarrow$ RestoreObservedAtoms($Y, X$)
      $Y \leftarrow$ IdealTemplateFallback($Y$) for atoms with $W_{r,a} = 0$
  end if
  $Y \leftarrow$ RefineGeometryAndClashes($Y, n_{\text{refine}}$, use_cssr)
  return $Y$
```

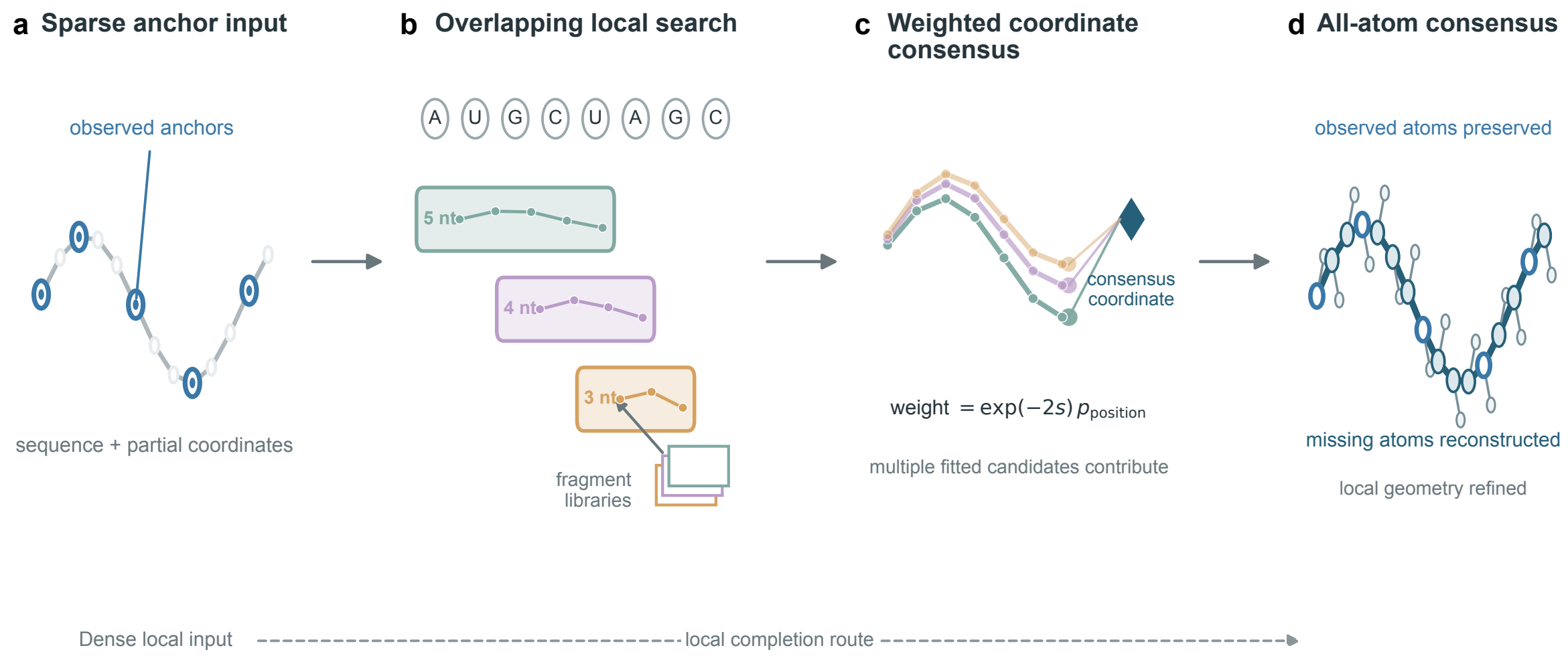

Supplementary Figure S1: Mechanistic overview of RAFA all-atom structure reconstruction. (**a**) Sparse input provides the RNA sequence and observed anchor coordinates. (**b**) Overlapping 5-, 4-, and 3-nt windows retrieve experimentally observed all-atom fragments from the core library and, when selected by the user, one category-matched expert library. (**c**) Candidate fragments are rigidly fitted and scored, and the retained candidates contribute to atom-specific coordinate accumulators with score- and window-position-dependent weights. (**d**) Weighted coordinate consensus reconstructs missing atoms while preserving observed input atoms, followed by local geometric refinement. The dashed lower path denotes local atom completion for information-rich inputs.

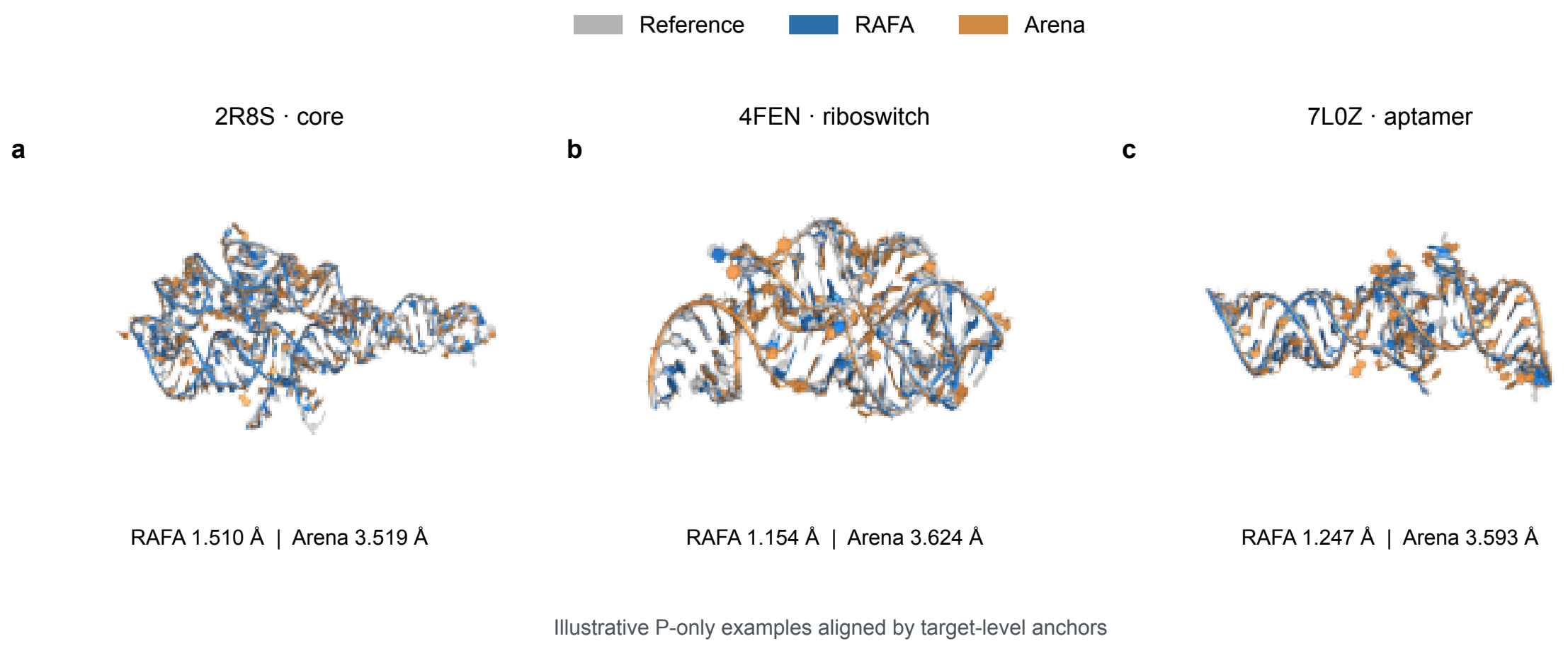

Supplementary Figure S2: Structural superpositions of the reference structures and P-only reconstructions for three illustrative targets from the original benchmark: (**a**) core RNA 2R8S, (**b**) riboswitch 4FEN, and (**c**) aptamer 7L0Z. Reference structures are shown in grey, RAFA reconstructions in blue, and Arena reconstructions in orange. For each target, both reconstructions were aligned to the reference using the target-level anchor-alignment procedure employed in the quantitative evaluation and were rendered in PyMOL using identical settings. The all-atom RMSDs for RAFA and Arena were 1.510 and 3.519 Å for 2R8S, 1.154 and 3.624 Å for 4FEN, and 1.247 and 3.593 Å for 7L0Z, respectively.

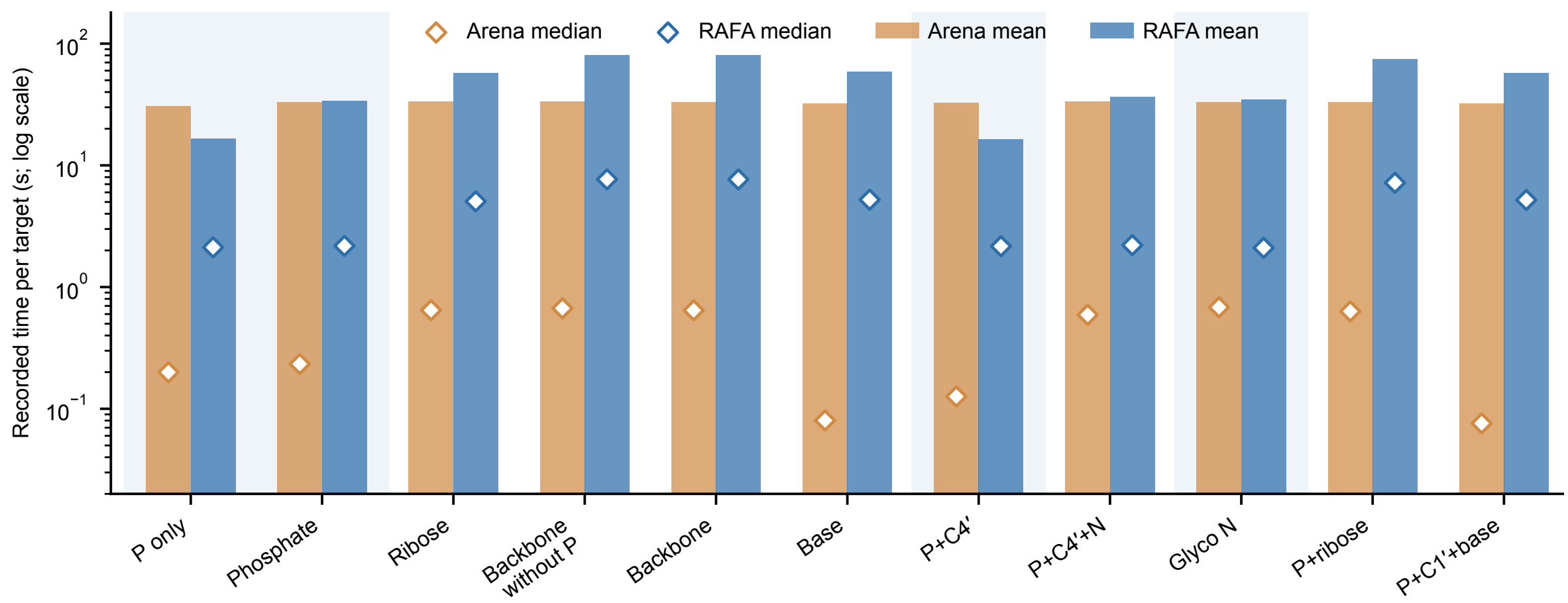

Supplementary Figure S3: Recorded runtimes across 72 targets and 11 input modes in the original benchmark. Bars show mean recorded time per target, diamonds show medians, and the vertical axis uses a logarithmic scale. Several large RNA targets increased the mean Arena runtime.

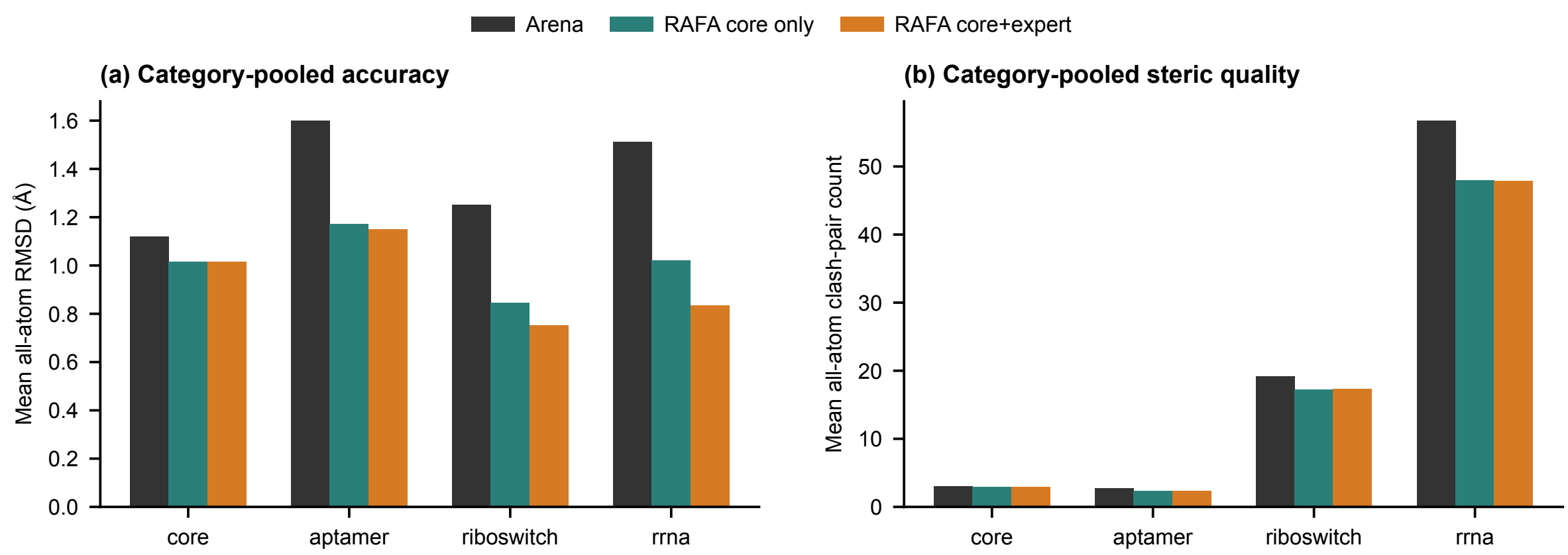

Supplementary Figure S4: Category-pooled performance on the original 72-target benchmark. (**a**) Mean all-atom RMSD. (**b**) Mean all-atom clash-pair count. Values are pooled descriptively across 11 input modes and stratified by target category: core RNA ($n = 44$), aptamer ($n = 8$), riboswitch ($n = 9$), and rRNA ($n = 11$). Bars compare Arena, core-only RAFA, and category-informed core+expert RAFA.

Supplementary Tables

Supplementary Table S2: Additional RNA 3D Hub equivalence-class representatives from test PDB entries that remained in the original source pool.

Remaining equivalence class	Library category	Shared test PDB
NR_4.0_76090.17	rRNA	9N5T
NR_4.0_97539.15	rRNA	9N5T
NR_4.0_92130.5	core	9N5T
NR_4.0_85511.1	rRNA	9ZNG
NR_4.0_44034.1	rRNA	9ZNG
NR_4.0_31579.1	rRNA	9TVU
NR_4.0_22916.1	rRNA	9TVU
NR_4.0_20488.1	core	9TVU
NR_4.0_10157.169	rRNA	8B0X
NR_4.0_33017.1	core	7P3K

Supplementary Table S3: Original 72-target per-mode all-atom RMSD comparison. Δ is RAFA minus Arena. P values are two-sided paired Wilcoxon signed-rank tests with Holm correction across 11 modes.

Mode	RAFA	Arena	Mean Δ	Median Δ	IQR Δ	Lower/tie/higher	Holm P
P only	1.852	3.024	−1.173	−1.084	[−1.969, −0.479]	66/0/6	9.38×10^{-11}
Phosphate only	1.326	2.043	−0.717	−0.618	[−1.347, −0.190]	61/0/11	1.32×10^{-7}
Ribose only	0.933	0.834	+0.100	+0.091	[−0.004, +0.146]	24/0/48	1.22×10^{-7}
Backbone without P	0.673	0.681	−0.008	−0.013	[−0.019, −0.001]	56/0/16	4.76×10^{-5}
Backbone only	0.669	0.677	−0.008	−0.012	[−0.020, −0.000002]	54/2/16	8.60×10^{-5}
Base only	0.788	0.833	−0.045	−0.147	[−0.356, −0.004]	54/0/18	4.42×10^{-4}
P + C4′	1.178	2.050	−0.872	−0.851	[−1.527, −0.141]	65/0/7	1.36×10^{-9}
P + C4′ + N	0.722	0.576	+0.146	−0.048	[−0.170, +0.011]	50/0/22	0.0272
Glycosidic N only	1.231	1.768	−0.537	−0.565	[−1.006, +0.003]	53/1/18	1.01×10^{-6}
P + ribose	0.801	0.761	+0.039	−0.004	[−0.013, +0.066]	44/1/27	0.649
P + C1′ + base	0.501	0.499	+0.003	−0.002	[−0.015, +0.011]	37/0/35	0.884

∞

Supplementary Table S4: RNA3DB 275-target per-mode all-atom RMSD comparison. Δ is RAFA minus Arena. P values are two-sided paired Wilcoxon signed-rank tests with Holm correction across 11 modes.

Mode	RAFA	Arena	Mean Δ	Median Δ	IQR Δ	Lower/tie/higher	Holm P
P only	2.672	4.031	-1.359	-1.374	$[-1.582, -1.073]$	274/0/1	8.24×10^{-46}
Phosphate only	1.951	2.790	-0.839	-0.828	$[-1.070, -0.584]$	267/0/8	2.83×10^{-45}
Ribose only	1.177	1.051	$+0.127$	$+0.117$	$[+0.081, +0.170]$	10/0/265	1.71×10^{-45}
Backbone without P	0.900	0.923	-0.023	-0.022	$[-0.032, -0.015]$	270/0/5	1.55×10^{-43}
Backbone only	0.901	0.925	-0.024	-0.022	$[-0.032, -0.015]$	273/0/2	8.24×10^{-46}
Base only	0.824	1.031	-0.207	-0.189	$[-0.288, -0.108]$	254/0/21	1.18×10^{-42}
P + C4′	1.446	2.627	-1.181	-1.210	$[-1.479, -0.881]$	275/0/0	8.24×10^{-46}
P + C4′ + N	0.763	0.680	$+0.083$	$+0.063$	$[-0.026, +0.186]$	91/0/184	2.61×10^{-13}
Glycosidic N only	1.389	2.131	-0.742	-0.712	$[-0.958, -0.510]$	268/0/7	1.82×10^{-45}
P + ribose	0.999	0.970	$+0.029$	-0.014	$[-0.025, +0.081]$	173/0/102	0.0476
P + C1′ + base	0.547	0.530	$+0.018$	$+0.015$	$[+0.001, +0.035]$	62/0/213	3.25×10^{-24}

Supplementary Table S5: Generic PDBFixer completion baseline on the original benchmark. NA indicates that sparse-input runs did not yield comparable evaluated reconstructions for aggregation.

Input mode	Arena RMSD	RAFA RMSD	PDBFixer ok/n	PDBFixer RMSD	PDBFixer clashes
P only	3.024	1.852	71/72	NA	NA
Phosphate only	2.043	1.326	71/72	NA	NA
P + C4′	2.050	1.178	71/72	NA	NA
P + C4′ + N	0.576	0.722	71/72	NA	NA
Glycosidic N only	1.768	1.231	71/72	NA	NA
Base only	0.833	0.788	72/72	1.670	803.583
P + C1′ + base	0.499	0.501	72/72	1.067	317.556
Ribose only	0.834	0.933	72/72	1.844	810.347
P + ribose	0.761	0.801	72/72	1.836	729.444
Backbone without P	0.681	0.673	72/72	2.278	678.792
Backbone only	0.677	0.669	72/72	2.280	656.597

Supplementary Table S6: Descriptive original-benchmark summary. Sparse 4 comprises P-only, phosphate-only, P+C4′, and glycosidic-N-only modes. Residue coverage replaces the previous ambiguous term atom coverage.

Subset	Method	RMSD (Å)	MAE (Å)	Residue coverage	Clash pairs
All 11	RAFA core+expert	0.970	0.314	0.989	11.542
All 11	RAFA core only	1.013	0.327	0.989	11.554
All 11	Arena	1.250	0.399	0.989	13.264
Sparse 4	RAFA core+expert	1.397	0.503	0.972	0.160
Sparse 4	RAFA core only	1.500	0.536	0.972	0.188
Sparse 4	Arena	2.221	0.758	0.972	3.736
Remaining 7	Arena	0.694	0.193	0.999	18.708
Remaining 7	RAFA core+expert	0.727	0.205	0.999	18.046

Supplementary Table S7: Illustrative P-only structural-superposition examples. Improvement is defined as Arena RMSD minus RAFA RMSD.

Target	PDB	Category	Length	RAFA	Arena	Improvement
NR_4.0_39627.8	2R8S	core	159	1.510	3.519	2.008
NR_4.0_89429.8	4FEN	riboswitch	67	1.154	3.624	2.469
NR_4.0_57264.3	7L0Z	aptamer	69	1.247	3.593	2.346

Supplementary Notes

All numerical summaries were regenerated from the retained per-target evaluation files. The RNA3DB sequence-identity and paired-analysis outputs are distributed with the manuscript assets, together with the original per-target benchmark outputs and generation scripts.
